# Genome characterization and CRISPR-Cas9 editing of a human neocentromere

**DOI:** 10.1007/s00412-022-00779-y

**Published:** 2022-08-17

**Authors:** Antonio Palazzo, Ilaria Piccolo, Crescenzio Francesco Minervini, Stefania Purgato, Oronzo Capozzi, Pietro D’Addabbo, Cosimo Cumbo, Francesco Albano, Mariano Rocchi, Claudia Rita Catacchio

**Affiliations:** 1 Department of Biology, University of Bari Aldo Moro, Bari, Italy; 2 Department of Emergency and Organ Transplantation (D.E.T.O.), Hematology and Stem Cell Transplantation Unit, University of Bari Aldo Moro, Bari, Italy; 3 Department of Pharmacy and Biotechnology, University of Bologna, Bologna, Italy

**Keywords:** Neocentromere, CRISPR-Cas9, Long-read sequencing, Isochromosome

## Abstract

The maintenance of genome integrity is ensured by proper chromosome inheritance during mitotic and meiotic cell divisions. The chromosomal counterpart responsible for chromosome segregation to daughter cells is the centromere, at which the spindle apparatus attaches through the kinetochore. Although all mammalian centromeres are primarily composed of megabase-long repetitive sequences, satellite-free human neocentromeres have been described. Neocentromeres and evolutionary new centromeres have revolutionized traditional knowledge about centromeres. Over the past 20 years, insights have been gained into their organization, but in spite of these advancements, the mechanisms underlying their formation and evolution are still unclear. Today, through modern and increasingly accessible genome editing and long-read sequencing techniques, research in this area is undergoing a sudden acceleration. In this article, we describe the primary sequence of a previously described human chromosome 3 neocentromere and observe its possible evolution and repair results after a chromosome breakage induced through CRISPR-Cas9 technologies. Our data represent an exciting advancement in the field of centromere/neocentromere evolution and chromosome stability.

**Supplementary Information:**

The online version contains supplementary material available at 10.1007/s00412-022-00779-y.

## Introduction

The centromere is a vital chromosomal structure for all living cells since it guarantees the faithful segregation of the genetic material during mitotic and meiotic cell divisions. More than 130 years of studies agree with the pivotal role of the centromere in sister chromatid cohesion or release and in the modulation of spindle dynamics, thus orchestrating the ordered movement of chromosomes to daughter cells (McKinley and Cheeseman 2016).

For a long time, what seemed to be fundamental to fulfill these tasks at a sequence level was having a conspicuous number of repeats organized in tandem arrays, as attested by the great abundance of repetitive DNA sequences in virtually all eukaryotic centromeres (corresponding from 5 to 50% of the entire chromosome) (Choo 1997). Humans are no exception since human centromeres are C-banding-positive and typically carry as many as 2–4 Mb of a 171-bp monomer, repeated in a head-to-tail fashion, known as α-satellite (Tyler-Smith and Brown 1987).

The relationship between DNA sequence and centromere function became less and less compelling after the discovery, in 1993, of a marker chromosome devoid of any alphoid monomer (Voullaire et al. 1993) but still able to bind all known essential centromere proteins and performing identically to its satellite DNA-based counterparts in mitosis and meiosis. This unexpected case of neocentromerization proved that the presence of α-satellite is neither a necessary nor a sufficient condition for centromeric activity in humans, thus challenging what we have always believed to know about centromeres and their functioning.

The observation of human centromeres in ectopic positions became more and more frequent, and most of them have been ascertained either at prenatal diagnosis or by cytogenetic analysis of individuals with congenital abnormalities, developmental delay, or intellectual disability (Amor and Choo 2002). In all cases, karyotyping revealed the presence of a small supernumerary marker chromosome (sSMC), lacking any detectable α-satellite but still mitotically stable due to neocentromerization. This phenomenon is probably the result of an ultimate rescuing mechanism by which a cell can retain a terminal or an interstitial acentric fragment deriving from single- or double-strand breaks in the same chromosome arm (Poot 2017). According to clinical records, the most common mechanism to prevent genetic material loss is the de novo inverted duplication (InvDup) of a distal acentric fragment (Amor and Choo 2002) or the creation of isochromosomes (Dalton et al. 1998; Izumi and Krantz 2014; Mertens et al. 1994), having two copies of a chromosome segment oriented as a “mirror image” straddling the breakpoint. The concurrent activation of a neocentromere at an interstitial site guarantees the ultimate meiotic/mitotic stabilization of the new sSMC therefore creating a trisomic or tetrasomic karyotype (Burrack and Berman 2012).

Noteworthy, not all cases of neocentromerization have been associated with chromosomal aberrations, karyotypic imbalance, and phenotypic abnormalities. Indeed, several independent works reported neocentromere emergence at euchromatic sites in the absence of any other detectable rearrangement (Amor et al. 2004; Bukvic et al. 1996; Capozzi et al. 2009; Rivera et al. 1996; Tyler-Smith et al. 1999; Ventura et al. 2004). All cases reported healthy individuals with cytogenetically normal and mitotically stable karyotypes, carrying a pseudo-dicentric chromosome containing an active neocentromere at an euchromatic region and a silenced alphoid centromere.

Human clinical cytogenetic data show a blatant non-random distribution of neocentromeres (arisen on both sSMCs and pseudo-dicentric chromosomes) since they preferentially cluster in specific regions of human karyotype, such as 3q, 8p, 9p, 13q, 15q, and Yq (Amor and Choo 2002). What makes a region more prone to be a “neocentromerization hotspot” is still unclear; the absence of any sequence recurrence in human neocentromeres supports the hypothesis that neocentromeres are epigenetically seeded (Tolomeo et al. 2017). For some reason, neocentromeric hotspots must be more permissive for particular epigenetic signatures, inducing nucleosome modifications (centromeric protein A, CENP-A, replaces the H3 histone at active centromeres), kinetochore assembly, and chromosome segregation machinery recruitment (Burrack and Berman 2012).

Nevertheless, a correlation between the regions frequently involved in human neocentromerization and the sites of centromere repositioning during primate evolution has been noticed: it seems that the inactivated domain of a centromere is a preferential seeding site for neocentromerization (Capozzi et al. 2009; Cardone et al. 2006; Ventura et al. 2003; Ventura et al. 2004). This seeming memory for ancestral centromere localization is apparently triggered neither by ancient alphoid debris nor by a specific DNA motif (Marshall et al. 2008).

We then thought of using a system in which acentric fragments could be created containing a normal centromere, which had become inactive due to a neocentromerization event along the chromosome itself. However, this is not feasible in normal mammalian cells because partial aneuploidy certainly undergoes counter-selection. We have therefore created interspecific cellular hybrids in which the permanence of human chromosomes is non-essential and therefore not counter-selected.

Even if neocentromerization has now long captured the attention of the scientific community, we still do not have a comprehensive and detailed knowledge of this phenomenon. To clarify when, how, and why a neocentromere arises, the observation of a “newly” formed centromere in an in vitro model would be extremely helpful.

Through the enormous strides in genomic engineering, the CRISPR/Cas9 machinery has been lately largely used for genome editing in cells and zygotes of different species (Cong et al. 2013; Hwang et al. 2013; Ménoret et al. 2015; Niu et al. 2014; Wang et al. 2013) and even for genomic rearrangement generation (Blasco et al. 2014).

To fulfill the abovementioned objectives, we created a human-hamster hybrid cell line containing a human pseudo-dicentric chromosome 3, starting from a lymphoblastoid cell line heterozygous for a neocentromere on chromosome 3 (Neo3) (Ventura et al. 2004). We were thus able to finely characterize this neocentromere by molecular methods, such as MinION sequencing, fluorescent in situ hybridization (FISH), and immuno-FISH experiments. Importantly, by using CRISPR/Cas9 technology, we also mimicked a chromosome arm breakage separating the neocentromere from the inactive canonical centromere 3 (Cen3).

Based on the abovementioned literature demonstrating an evolutionary memory for centromere signatures, we took up the challenge of testing and investigating the behavior and fate of an acentric fragment containing centromeric chromatin in an in vitro system. On the other side, we observed various healing rearrangements the other fragment bearing Neo3 underwent to stabilize himself.

## Methods

### Cell culture

All cell lines used in this work have been maintained with standard protocol and in accordance with the manufacturer’s instructions using RPMI media, implemented with 1% L-Gln and 1 × Pen-Strep. Culture conditions were 37 °C and 5% CO_2_.

### ChIP-on-chip analysis

Chromatin immuno-precipitation (ChIP) analysis was performed as previously described (Wells and Farnham 2002). Briefly, lymphoblastoid cells containing Neo3 were cross-linked in situ by adding formaldehyde to a 1% final concentration directly to the culture medium, and DNA was shared by sonication. Immunoprecipitation was performed using polyclonal antibodies against centromeric proteins CENP-A and CENP-C (Trazzi et al. 2009). Purified DNA fragments were amplified using the Whole Genome Amplification kit (Sigma-Aldrich). The labeled ChIP and total DNAs were co-hybridized to a NimbleGene Whole-Genome Tiling array (HG17Tiling Set 9, see Files S1 and S2 for details), which had an average resolution of 100 bp. DNA-binding peaks were identified by using the statistical model and methodology described at http://chipanalysis.genomecenter.ucdavis.edu/cgi-bin/tamalpais.cgi using stringent parameters for peak identification (98th percentile threshold and *p* < 0.0001) (Bieda et al. 2006).

### Hybrid cell line

Somatic cell hybrids were generated by fusing TK-Chinese hamster B14-150 (ATCC: CCL 14.1) cells with the human lymphoblastoid cell line containing the Neo3 reported in (Ventura et al. 2004) (case 2). HAT selection was applied to the medium the day after. Monoclonal colonies were isolated and seeded in 24-well chambers after 10 days. Alu-PCR amplification was performed on isolated hybrid DNAs as already described (Archidiacono et al. 1994) using the following Alu primers: 5′-GGATTACAGGYRTGAGCCA-3′; 5′-RCCAYTGCACTCCAGCCTG-3′ (Y = C/T; R = A/G). FISH experiments were performed using labeled Alu-PCR products on human metaphases to identify positive clones.

### Long-range polymerase chain reaction (PCR)

To characterize the nucleotide sequence of the neocentromeric region identified, a nanopore-based sequencing strategy was used, with the third-generation platform named MinION (Oxford Nanopore Technologies Inc.) (Magi et al. 2018). For this purpose, genomic DNA (gDNA) was isolated from 5 × 10^6^ cells using the Blood and Cell Culture DNA Mini Kit (Qiagen) and quantified with the Qubit 2.0 Fluorometer (Thermo Fisher Scientific). The region of interest (300 kb) has been amplified using 32 overlapping amplicons with long range PCR performed using LA Taq DNA polymerase (TaKaRa cat RR002M). The reaction was performed according to the manufacturer’s instructions. Each amplicon has been obtained with 30 cycles with the following conditions: 94 °C for 20″, 60 °C for 20″, and 68 °C 13′. The primers sequences are listed in Table 1. Amplicons of the expected size were cut and eluted from gels using the QIAquick Gel Extraction Kit (Qiagen). Before starting the library preparation, we quantified and estimated the purity of samples (Nanodrop, Thermo Fisher Scientific). The amplicons were then pulled to an equal weight ratio, and 1 μg of the pool was diluted to 45 μl in nuclease-free water and prepared for sequencing.

**Table 1:** Primer pairs used for Neo3 region amplification

Primer pair #	GRCh38/hg38 coordinates			Forward	Reverse
	chr	start	end		
1	chr3	147,420,925	147,430,740	CGTGATTTGGGTCCCTTTGTGTTTG	CAGCAGGCATAAGACAGTGATAAGC
2	chr3	147,430,526	147,441,641	TTCCTTCCCTGAGCTTGCTATAACC	CAGGCTAGTGTCCACATTTGTCTCT
3	chr3	147,441,081	147,453,599	GAGGGTTGTGTCCAGGAAGTCATAT	AGCCAAAGTCCTCACAACACCTACA
4	chr3	147,453,364	147,465,886	AGGGAGGACTCAATCAGAAGGTAAC	TGCTTAGCCTATTCTGCCACTCTTG
5	chr3	147,465,417	147,475,956	GACGATTGGAGTAAGTTGCTGAGGA	TCCCTGCGTTTGGCTAGTAAGAGAA
6	chr3	147,473,577	147,486,159	GGCTGAGAAATTCCTTAGGGTACAC	CTGGAGAGGATGTGGAGAAATAGGA
7	chr3	147,483,667	147,493,732	GCACTGGCTGTTATGAACCTCAAAG	CCACCTAAATGCCATCTCACCACTA
8	chr3	147,492,890	147,502,575	TTTCCAACACAGCCATGCCTTCTCT	CCTTTCCTCCATACTCTAGCTCACA
9	chr3	147,501,696	147,511,790	TGGCATCCAGTAGAGAAGCAACAAC	TCCTGGGACACGATGAATAGAACAC
10^a^	chr3	147,510,925	147,516,451	GGAAAGTACTGGGTTGGAAGATCAC	GAGAATTACTTCAACCAGGGAGGTG
11	chr3	147,515,711	147,527,560	TGGCCATATTAAAATTGAGAGGTTG	AGCTCTACAAGCCAAAAGAGATTGG
12	chr3	147,524,638	147,534,476	GGGATAGTTTGACTCCCTCTCTTCC	AACAAAAGCCAATTTTGCCATAGTC
13	chr3	147,533,590	147,543,752	TGCCAAAGTTGTTCACATTTACTCTC	ACATTTACCTCCCATTCACATCTCC
14	chr3	147,533,953	147,544,416	CTCCAGAAAGTTCCCACATACACCT	ACTGCTAACCCTCACCCATCAATGT
15	chr3	147,544,021	147,554,893	GCCTGGAGAAGAAGCCTTGTTGTTT	ATGCCAGGATCTGTTGACAAGTCAC
16	chr3	147,554,217	147,566,140	CAAGGTCACAACCAGCTAAGAGTAG	TGGTGTCCTTGTCTGGTTTGGGTAT
17	chr3	147,564,520	147,574,790	CACCAGACCCAAACAACAAAGAGGA	GTGCTGAGAGACTGTGACATTGATG
18	chr3	147,573,415	147,583,817	CCAGAGATGACCTTGAGGTTTGAGA	GGGCTTCTACTGTCTTTCTGCTTTC
19	chr3	147,579,858	147,590,029	AGTAAGGGCAGTGTAGCCACAGAAG	GGCTGATTCCAGAACTACCTTCATC
20	chr3	147,589,137	147,599,282	AGTAGGCAATGGAGGAAGTGAGAAG	CTGAGTAGCCTAACTGGGAAACACC
21	chr3	147,598,839	147,610,214	CTCGTCAAAGTCATTCTCCATCAAG	TCAGAAGTGAATGTTCCATGATTCC
22^b^	chr3	147,609,648	147,618,987	GTTCACGTTATTCTCCTGCCTCAG	ACCATTAACAATTTTCCGACTGGAG
23	chr3	147,614,059	147,625,695	GCCTAGCAGTTGGTGGGAGATTAAA	ATGCTTATGCTGGTCACCTTGTCCT
24	chr3	147,625,323	147,636,852	GCTGGGTAGTGACACTTGTTCTCTT	AGTGCTGACTTGGTAGTGGTGAATC
25	chr3	147,635,820	147,646,310	GGGAGAGTACCAAATCAAGGGATCA	GGTGGAAGAGAGGTAAAGAAGGAGA
26	chr3	147,644,824	147,656,848	GGCACCCAGTGAAAGCATATCGTAT	CTGGAAATGGGAGGAAGGATAAGAG
27	chr3	147,656,177	147,667,880	GGGCACCACAGGTATGTTCATATCA	GGGAGGAGTGCTATGGTTTGAAGAT
28	chr3	147,667,241	147,678,552	TTGGGTTAAGGGATGCCTACATAGC	GAAGGAAGGAGGGAGAAAGGAAGTA
29^a^	chr3	147,678,135	147,689,849	GGCAGGACATTGAAGAGTTGGTAGA	GCATTCTACCACCTCTCTCAACCTT
30	chr3	147,689,184	147,700,956	CCCATAGACCCTAACTCCAAACACT	GAGGGAATGGGAGAGGTTAAAGATG
31	chr3	147,699,438	147,709,847	AGGAAACGGATCTCTGTCTTGTGTG	CCAAGGCTAAACCAGGAAGAAGTCT
32	chr3	147,709,214	147,720,842	CTTCCATCTTGTGCCCACTCAGTAT	TAGGGATCTGCTGGGACTTTAGTCT

^a^Unresolved sequence

^b^Amplification not obtained

### Library preparation and MinION sequencing

According to the Ligation Sequencing Kit 1D (SQK-LSK108) protocol, the amplicons were end-prepared with the NEBNext Ultra II End Repair/dA-Tailing Module (New England Biolabs Inc.) and ligated to nanopore-specific adapters with Blunt/TA Ligase Master Mix (New England Biolabs Inc.). All purifications and the final library elution were performed with AgencourtAMPure XP beads (Beckman Coulter Inc.). After the Platform QC run and the priming of the flow cell, the sequencing mix was loaded and the NC_48Hr_sequencing_FLO-MIN107_SQK-LSK108 protocol was started (MinIONflowcell: FLO-MIN107). The sequencing run was stopped after 24 h.

### Sequence analysis

The fast5 files resulting from the sequencing were base-called using the Guppy algorithm. Fastq reads of at least 9 kb were then aligned on the GRCh38 human reference genome using minimap2 (Li 2018) with specific Nanopore platform parameters, and BAM files were visualized with the Integrative Genomics Viewer (IGV) browser (Robinson et al. 2011). Reads mapping at chr3:147,400,000–147,750,000 (GRCh38/hg38) were selected by samtools and were used for contig construction by canu assembler (Koren et al. 2017). Contigs were further corrected by Medaka consensus tool. Final contigs were then aligned on GRCh38 human reference genome using blat to verify the width of coverage and the similarity. The Augustus tool (Galaxy workbench) has been used for genome annotation while repeat sequences have been found using RepeatMasker free available software (www.repeatmesker.org).

### CRISPR-Cas9 mutagenesis

Cas9-mediated mutagenesis was performed using the expression vector pX333 (Addgene Plasmid #64,073 (Maddalo et al. 2014)). The vector expressing Cas9 was digested with BsaI restriction enzyme and ligated to annealed and phosphorylated oligonucleotides sgRNA_BsaI_F/R, targeting the Neo 3 pericentromeric region between nucleotides 147,103,679–147,103,698 (File S3).

The pX333_Bsa_sgRNA1 was modified by introducing in NotI restriction site the Neomycin cassette, amplified using the primers Neo_NotF/Neo_NotR (File S3). Cell Nucleofector 4D (Lonza) has been used to nucleofect 1 μg of pX333_NEO_BsaI_sgRNA1 in Hy_Neo3_A2 cell line according to the optimized provided protocol by manufacturer (Amaxa Biosystem, Cologne, Germany, www.amaxa.com). Briefly, cells were gently resuspended in 100 μl of the total volume of SF Cell Line 4D-Nucleofector Solution (Amaxa Biosystem), mixed with 1 ug of pX333_NEO_BsaI_sgRNA1 plasmid and pulsed with the program EO-100. Immediately after, cells were transferred into pre-warmed fresh medium in a 100-mm dish. Enrichment of transfected cells was done by G418 selection: the day after the medium was replaced with a fresh medium supplemented with G418 sulfate antibiotic (400 μg/ml) and cells kept under selection for 10 days. Twenty colonies were picked, transferred in a 25-cm^2^ cell culture flasks, and expanded in neomycin-free medium. FISH experiments have been performed to verify the integrity of chromosome 3.

GeneArt Genomic Cleavare Detection kit was used to perform the T7E1 assay according to the manufacturer’s instructions. The primers used are listed in File S3. The band quantification was performed with the Image Lab Software.

### FISH and immunoFISH

Metaphase spreads and interphase nuclei were obtained from the selected hybrid cell line. FISH experiments were performed using 13 human BAC clones (File S4) directly labeled by nick-translation with Cy3-dUTP, Cy5-dUTP, and fluorescein-dUTP (Enzo) as described by (Lichter et al. 1990), with minor modifications. Briefly, 300 ng of the labeled probe were used for the FISH experiments; hybridization was performed at 37 °C in 2 × SSC, 50% (v/v) formamide, 10% (w/v) dextran sulfate, 3 μg C0t-1 DNA, and 3 mg sonicated salmon sperm DNA, in a volume of 10 μl. Post-hybridization washing was at high stringency conditions: at 60 °C in 0.1 × SSC (three times). The nuclei and chromosome metaphases were simultaneously DAPI-stained. Digital images were obtained using a Leica DMRXA2 epifluorescence microscope equipped with a cooled CCD camera (Princeton Instruments). DAPI, Cy3, Cy5, and fluorescein fluorescence signals, detected with specific filters, were recorded separately as grayscale images. Pseudocoloring and merging of images were performed using the Adobe Photoshop software. Immunofluorescence using CENP-C antibody was performed as previously described (Earnshaw and Tomkiel 1992) with minor modifications. Metaphase preparations were stored in a fixative solution (methanol and acetic acid, 3:1) at − 20 °C, and few drops were used to prepare each slide. As soon as the surface was dry, each slide was rehydrated by immersion in 1 × PBS-Azide (10 mM NaPO4 at pH 7.4, 0.15 M NaCl, 1 mM EGTA, 0.01% NaN3) for 15 min at RT. Chromosomes were then swollen by washing the slides (three times, 2 min each) with 1 × TEEN (1 mM treithanolamine-HCl at pH 8.5, 0.2 mM NaEDTA, 25 mM NaCl), 0.5% Triton X-100, 0.1% BSA. The primary polyclonal antibody against the centromeric protein CENP-C was diluted 1:40 in the same solution and then added (100 μl) on the slides. Each slide was incubated for 2 h at 37 °C. Excess of primary antibody was removed by washing the slides at RT (three times: 2, 5, and 3 min each) with 1 × KB buffer (10 mM Tris–HCl at pH 7.7, 0.15 M NaCl, 0.1% BSA). Secondary antibody conjugated with FITC was diluted 1:40 in the same solution, and 100 μl was then added to the slides that were then incubated 45 min at 37 °C in a dark chamber. Following incubation with the secondary antibody, the slides were washed once with 1 × KB for 2 min, prefixed with 4% paraformaldehyde in 1 × KB for 45 min, washed with distilled H_2_O by immersion for 10 min at RT, and fixed with methanol and acetic acid (3:1) for 15 min. After that, FISH was performed following the standard procedure.

## Results

### A human-hamster hybrid cell line allowed the isolation of the Neo3

The patient carrying the identified neocentromere was found to be heterozygous for the neocentromere on chromosome 3 ((Ventura et al. 2004)). Therefore, a hybrid cell line containing the chromosome of interest (Neo3) in the absence of the wild-type homolog was created to study the neocentromere characteristics more easily.

All the 30 successfully dividing hybrid cell clones were tested cytogenetically to choose the one that mostly fulfilled our aims: we used a plasmid clone containing the alpha-satellite array from human chromosome 3, and we obtained four hybrid clones containing the Neo3 chromosome (named A2, A5, C1, and D2). The clone HY-NEO3-A2, containing human chromosomes 7, 8, 12, 13, 18, 19, 21, and Neo3, was then selected for the following experiments.

### ChIP-on-chip finely localized the Neo3 and showed a major and two minor CENP-A/CENP-C-enriched domains

**Fig. 1:**
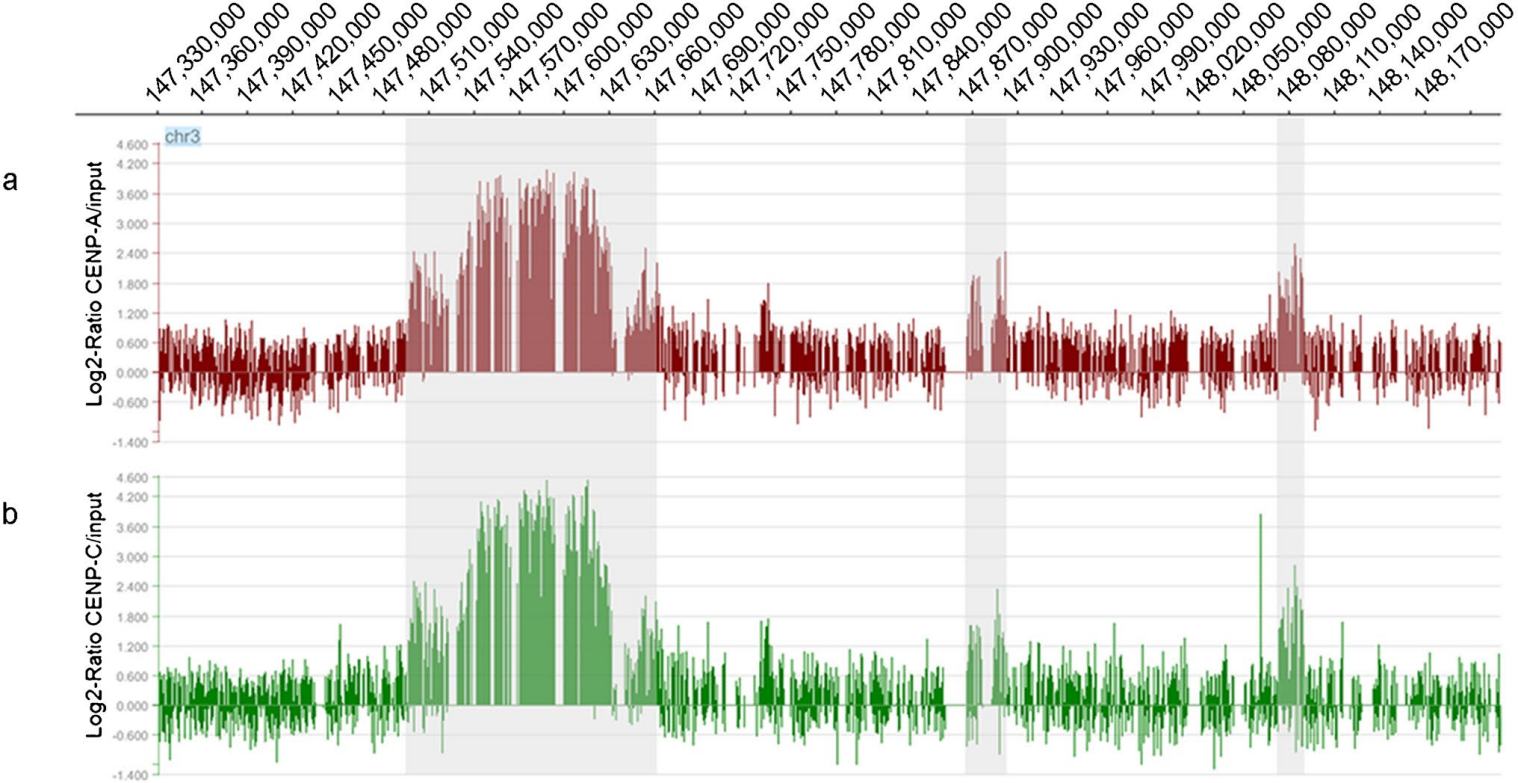
Partial view of the ChIP-on-chip analysis data on chromosome 3, using anti-CENP-A and anti-CENP-C antibodies. The results are presented as the log2 ratio between the hybridization signals obtained with immunoprecipitated DNA using anti-CENP-A (**A**) and anti-CENP-C (**B**) antibodies and that from the input DNA sample. The *X*-axis shows the genomic position of each oligo on chromosome 3. The data are visualized through the SignalMap software (NimbleGene Systems, Inc.). The shaded regions indicate the mapping of the CENP-A and CENP-C domains as identified by the Tamalpais statistical analysis: a major domain of about 163.6 kb (chr3:147,497,420–147,661,009, GRCh38/hg38) and two minor domains of about 21.5 and 6.7 kb (mapping at chr3:147,869,478–147,890,981 and chr3:148,079,102–148,085,846, GRCh38/hg38)

The position of the neocentromere at the sequence level, was assessed by performing ChIP-on-chip experiments using two rabbit polyclonal antibodies directed against CENP-A or CENP-C human centromeric proteins. The immunoprecipitated and purified DNA was amplified and hybridized to a NimbleGene Whole-Genome Tiling array, which has an average resolution of about 100 bp. The enrichment of ChIP DNA, before and after amplification, was validated by real-time PCR. The analysis showed that the CENP-A/CENP-C-associated chromatin is discontinuous, consisting of a major domain of about 163.6 kb and two minor domains of about 21.5 and 6.7 kb (Fig. 1).

### Sequence analysis showed no peculiar signatures on the Neo3

To investigate the genomic sequence of the neocentromere region, and highlight specific features linked to the seeding of a functional centromere, we divided the neocentromere region (~ 300 kb) in 32 partially overlapping amplicons sequenced by a long-read approach. Pooled amplicons were sequenced on a Oxford Nanopore’s MinION device and a total of 1,667,063 reads were produced of which 1,662,629 were *pass* and 4434 were *fail*. The obtained sequence covered the 94% of the target region, leaving about 18 kb unresolved (Table 1). *Pass* reads were used to produce an assembly made of 14 contigs that was aligned against the human reference genome and showed an identity of 99.9 to 100% (Tables 1 and 2).

**Table 2:** Sequence contigs details and identity scores

Contig	GRCh38/hg38 coordinates			Contig size (nt)	Matches (nt)	Identity (%)
	chr	Start	End			
tig00000008:25–9733	chr3	147,420,923	147,430,741	9818	9605	99.90
tig00000006:25–22,894	chr3	147,430,524	147,453,599	23,075	22,641	99.90
tig00000001:24–12,431	chr3	147,453,363	147,465,886	12,523	12,282	99.90
tig00000002:22–20,543	chr3	147,465,415	147,486,159	20,744	20,293	99.90
tig00000002:0–37,706	chr3	147,473,574	147,511,767	38,193	37,240	99.90
tig00000004:15–28,426	chr3	147,515,710	147,544,416	28,706	28,092	100.00
tig00000001:0–40,493	chr3	147,544,031	147,585,181	41,150	39,857	99.90
tig00000001:4772–25,626	chr3	147,573,414	147,594,456	21,042	20,605	99.90
tig00000002:22–11,279	chr3	147,598,838	147,610,214	11,376	11,145	99.90
tig00000009:24–11,511	chr3	147,614,056	147,625,695	11,639	11,331	99.90
tig00000003:12,084–52,581	chr3	147,625,333	147,666,291	40,958	40,012	99.90
tig00000001:20–22,142	chr3	147,656,174	147,678,552	22,378	21,872	100.00
tig00000002:22–11,630	chr3	147,689,181	147,700,956	11,775	11,488	100.00
tig00000005:1019–31,385	chr3	147,690,195	147,720,842	30,647	29,994	99.90

The sequenced region turned out to be a gene desert like the reference region. It resulted to be an AT-rich region with a comparable base composition to the wild-type region (64.41% and 64.58%, respectively). Repeated element distribution is summarized in File S5.

### CRISPR-Cas9 mediated different chromosome rearrangements

**Fig. 2:**
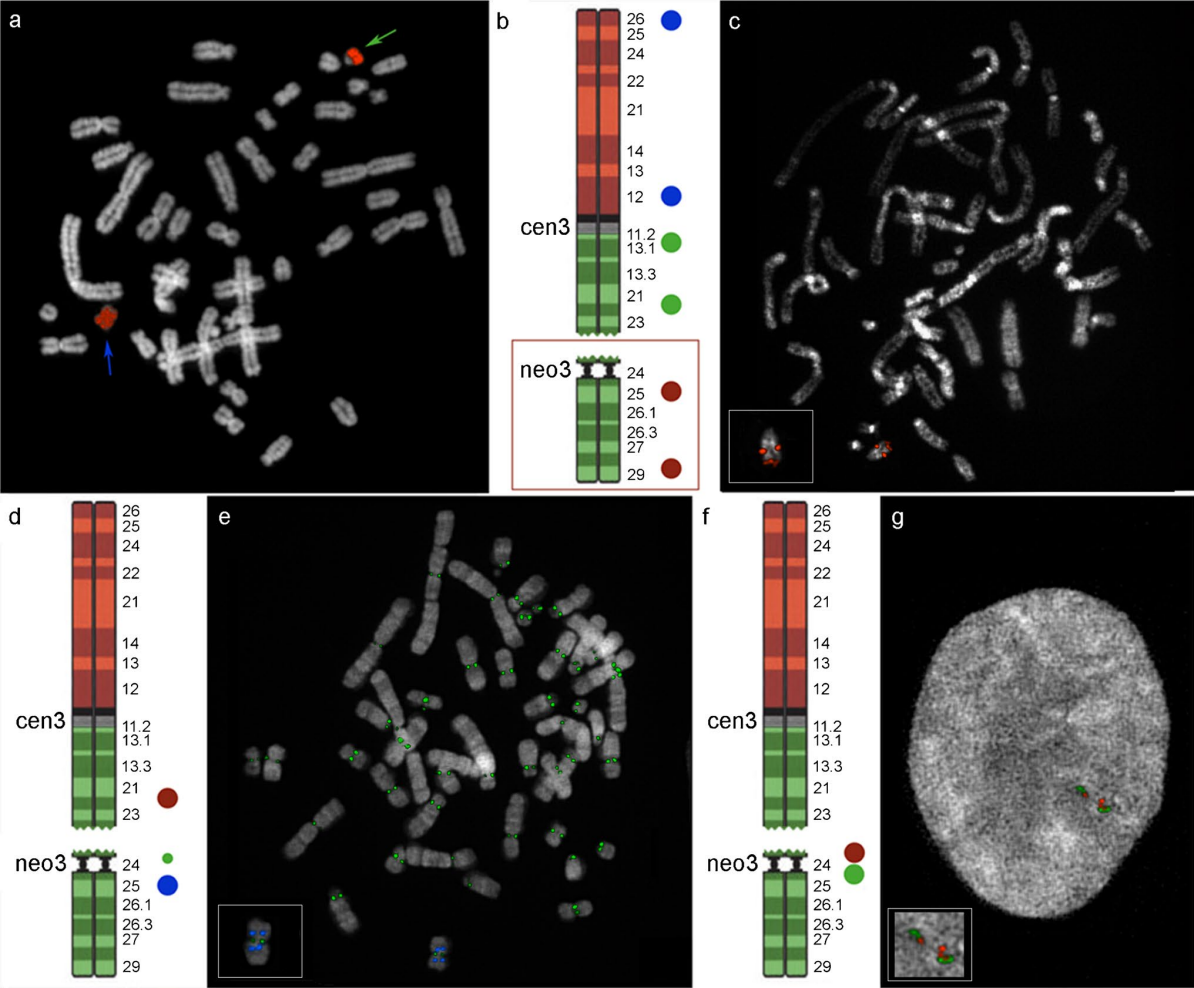
FISH and immuno-FISH results. **a** Hybridization result obtained by using as a probe a chromosome 3 WCP. A small acrocentric chromosome (green arrow) and a small metacentric chromosome (blue arrow) are visible in the panel. **b** Probe selection for the cytogenetic characterization through BAC clone hybridization (blue probes: RP11-151A4 and RP11-655A17; green probes: RP11-454H13 and RP11-21N8; red probes: RP11-498P15 and RP11-693H4). **c** An exemplifying result of the hybridization performed using the probes described in **b**.** d** Probe selection for the immuno-FISH characterization (red probe: RP11-21N8; green: anti-CENPC antibody; blue probe: RP11-498P15). **e** FISH result of the hybridization performed using probes described in **d**. **f** Probe selection for the FISH characterization of the region flanking the break site (RP11-426P6 in red and RP11-1077E14 in green). **g** Interphase FISH result of the experiment described in **f** showing the inverted duplication of the probes in one of the derived chromosomes. Extracted and enlarged chromosomes are shown on the bottom left of **c**, **e**, and **g** for better clarity

**Fig. 3:**
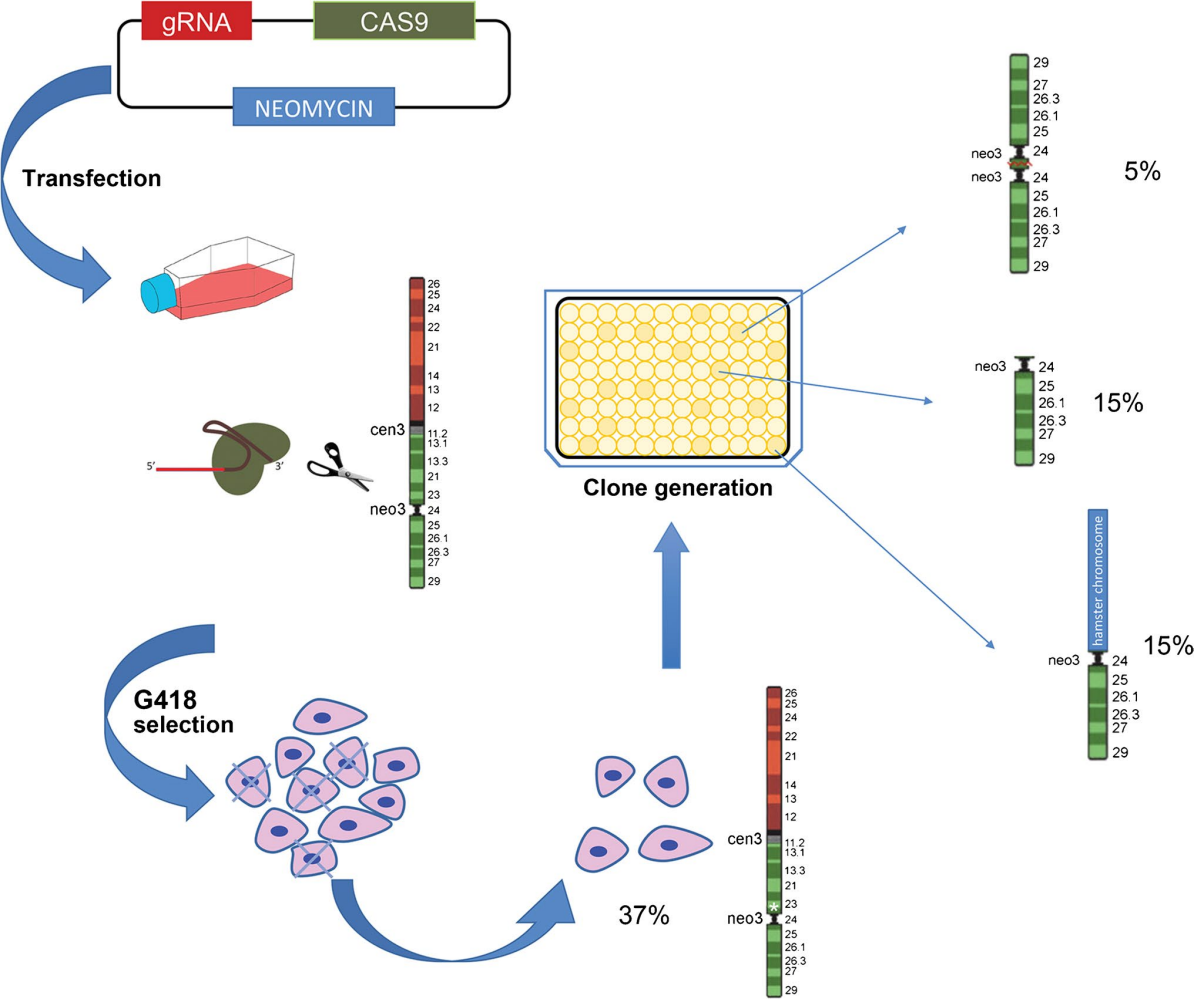
Schematic showing the editing with CRISPR-Cas 9. The white asterisk indicates small InDels obtained by NHEJ repair. The shown percentages illustrate the editing detectable events (37% of InDels and 35% of chromosomal rearrangements). Consequently, the remaining 28% consists either of non-edited cells or of undetectable mutations

The main purpose of the editing on chromosome 3 carrying the Neo3 concerns the observation of the fate of the two fragments produced in a low-pressure selective context such as that of a hybrid cell line. Our conceived strategy was aimed to test the “memory” of an inactivated canonical centromere and, at the same time, to witness possible healing chromosomal rearrangements that would give us precious pieces of information about the stability-seeking methods carried out by cells. We edited the hybrid cell line by inducing a breakage about 300 kb upstream to the major domain of the neocentromere (Figs. 2 and 3).

To minimize the elements that contribute to a lower editing efficiency, we created a plasmid in which, in addition to the gene for Cas9 and the gRNA, we cloned the neomycin resistance cassette. In this way, after transfection, we selected resistant cells, but without pushing selection to induce integration of the plasmid into the cell genome. Therefore, after a 10-day selection period, the cells were partly harvested and characterized by FISH and molecular biology techniques and partly used to generate cell clones.

Firstly, we used a human chromosome 3 whole chromosome painting (WCP) probe in a FISH experiment and revealed that 17/50 of the analyzed metaphases showed signs of the induced chromosomal breakage. The results showed small acrocentric chromosomes, small metacentric chromosomes (both visible in Fig. 2a), and fragments of human chromosome 3 fused with hamster chromosomes. Neither of these was appreciable before editing.

Most likely, the metaphases that showed the normal chromosome 3 carrying the neocentromere could be the result of one of the following destinies: (1) not have been affected by the editing process and (2) have experienced the breakage and repaired it in a way that could not be detected by cytogenetic approaches. To investigate the latter scenario, we performed the T7E1 assay measuring a 36.9% of short InDel mutations (File S6).

We isolated 20 single cell clones. A preliminary screening by PCR was performed with a pair of primers that amplify a 300-bp sequence straddling the guide location. Clones that gave a PCR band were considered WT or otherwise not carrying cytogenetically relevant rearrangements. Seven clones did not give an amplicon and a more in-depth cytogenetic analysis was performed on these. This produced the following results regarding the edited chromosome 3: no trace of the chromosome 3p terminal to 3q24 was found in all the analyzed cells, as revealed by the absence of blue and green probes in Fig. 2b, c; three clones showed a single small acrocentric chromosome (clones C8, D8, and G8) (Fig. 2b, c); three clones showed human/hamster fused chromosomes (clones A8, E8, and H8) (File S7), and one clone showed a small isochromosome (clone F8) (Fig. 2d, e).

Then, we performed an immuno-FISH assay to verify the centromeric functionality of the neocentromere in the derived chromosomes (Fig. 2d, e, and File S7). Surprisingly, clone G8, containing the 3q terminal fragment rescued as an acrocentric chromosome, showed a partial duplication of the BAC RP11-693H4 on its tiny p arm, revealing that a terminal duplication from the 3q telomere has occurred (File S8).

Lastly, we characterized the very vicinity of the breakage site in one of the derived chromosomes, by using interphase-FISH and revealed the inverted duplication of the analyzed region (Fig. 2f, g).

## Discussion

Neocentromeres and evolutionary new centromeres (ENCs), defined as repositioned centromeres fixed in primate species distinguishing orthologous chromosomes (Cardone et al. 2007; Rocchi et al. 2009), have been extensively characterized, and the existence of latent centromeres was also proposed as a possible reason for the emergence of neocentromeres (Ventura et al. 2003). Nevertheless, little is still known about the mechanisms underlying their formation and evolution and, more generally, data explaining the essential features of the centromeric function are scarce, mostly due to the highly repetitive nature of primate centromeres that has hindered accurate molecular characterizations of these regions for a long time (Murillo-Pineda and Jansen 2020). Recently, the first telomere-to-telomere assemblies of human chromosomes, which include repeated arrays at centromeres and pericentromeres, have been produced with novel breakthrough technologies, opening the field to previously impossible analyses (Logsdon et al. 2021; Miga et al. 2020). This will allow, for example, the detailed intra- and inter-species comparison of the genomic sequence, organization, and epigenetics of centromere, neocentromeres, and evolutionary new centromeres without the limit of the sequence gaps mostly caused by repetitive sequences.

With this work, we investigate two main aspects: (i) we characterize the sequence and organization of a region harboring a human neocentromere that is able to be inherited through successive generations, and (ii) we test how this chromosome would evolve after a chromosome breakage. The question we have attempted to answer is: is the canonical centromere pushed to a reactivation in a hybrid cell line? What is the fate of two chromosome fragments with an active and inactive centromere in a low selective pressure context?

Considering the abovementioned, starting with a stabilized cell line from a male fetus (Ventura et al. 2004) heterozygote for the Neo3, we created a somatic cell hybrid to genetically isolate Neo3 from Cen3, its wild-type homolog. This cell line represents a useful cellular model to study the peculiar characteristics underlying the formation and evolution (if followed over time) of a centromere. By means of a ChIP-on-chip experiment, we defined a segment of about 160 kb as the main region affected by centromeric repositioning (Fig. 1). Besides, we sequenced by long-read methods the main domain of the neocentromere and the flanking regions, for a total of about 300 kb, revealing no major differences from the human reference genome (GRCh38/hg38).

No structural variation is present in the assembly sequence. Both regions (Neo3 and the corresponding region on GRCh38/hg38) are gene deserts as it was quite expected. They show comparable features: both are AT-rich sequences and with a concentration of repeated elements above 40%. The base composition is a prerequisite for the establishment of a new centromere, since centromeric regions are AT-rich structures due to the presence of the alpha satellite. However, the Neo3 sequence, like that of almost all neocentromeres, is AT-rich but devoid of alpha-satellite and this demonstrates the importance of the epigenetic control. In this view, the sequencing of neocentromeres is a push towards the knowledge of the structural features underlying their formation. Interestingly, the number of LINE1 insertions is increased in the Neo3 sequence (File S5), in agreement with the view that L1 plays a role in regulating neocentromere activity (Chueh et al. 2009). However, the LINE1 family is reported to have insertion site preference in regions of constitutive AT-rich heterochromatin (Acosta et al. 2008; Marsano and Dimitri 2022; Waters et al. 2004). Probably, the higher number of elements found in the sequenced Neo3 region is a consequence of this aspect. In addition, the presence of these extra LINE-1 elements compared to the wild-type sequence enlarged the Neo3 region.

Therefore, our data consolidate the hypothesis that the neocentromere formation and, more generally, the centromeric function are essentially epigenetic, as previously postulated (Gary et al. 1997), but it also opens to currently purely speculative considerations on a possible structural role played by retrotransposons such as L1.

In the last decade, the introduction of editing methods such as CRISPR-Cas9 has provided an accessible tool for genome manipulation. Different genomic structural variations have been induced for very different purposes (Blasco et al. 2014), and neocentromere formation by deletion of the endogenous centromere has been induced in different model organisms such as *Schizosaccharomyces pombe*, *Candida albicans*, *Cryptococcus deuterogattii*, or chicken cells (Ishii et al. 2008; Ketel et al. 2009; Schotanus and Heitman 2020; Shang et al. 2010). Recently, CRISPR-Cas9 methods have been successfully used for the first time to induce the seeding of a neocentromere (on chromosome 4) in the complex context of human cells, by excising an 8-Mb centromeric region and thus providing an excellent system to study the chromosomal site “before” and “after” the centromere activation (Murillo-Pineda et al. 2021). Although the neocentromere region was gene poor, neither sequence nor transcription changes have been revealed at the seeding site after 200 cell divisions, indicating that the satellite acquisition observed at newly formed centromeres over the course of evolution takes much longer evolutionary times (Rocchi et al. 2012; Tolomeo et al. 2017).

We here apply the CRISPR-Cas9 technology and induce a peri-neocentromeric break in chromosome 3 to generate a large acentric fragment containing the inactivated Cen3 and a small 3q terminal section harboring Neo3.

Our results show that no reactivation of the canonical, alpha-satellite-rich Cen3 was induced to rescue this acentric fragment. Likely, although bearing the “memory” of an active centromere (Cardone et al. 2006; Ventura et al. 2003; Ventura et al. 2004), the absence of selective pressure played against it. Indeed, in a hybrid cell line, the human chromosomal content has no role in cell propagation, so the presence or absence of one or more chromosomes has no effect. The other side of the coin is that the absence of a homologous chromosome allowed the recovery of an apparently high number of cytogenetic rearrangements (7 out of 20 isolated clones, 35%) detectable only by cytogenetic methods (Rayner et al. 2019). Importantly, in order for a linear chromosome to be stable in a cell line, the presence of a functional centromere is not sufficient, since the existence of the two intact telomeres is essential. Therefore, linear chromosomes with terminal breaks are rescued if stabilized by further structural rearrangements (O’Sullivan and Karlseder 2010).

Our genome editing procedure allowed us to follow the destiny of the two derivative chromosomal fragments: an acentric, satellite-rich big piece of roughly 147 Mb, and a small (about 51 Mb) acrocentric fragment containing the neocentromere. Following the breakage, the biggest piece was totally lost, likely being able neither to repair the terminal damage, nor to activate any centromere. Instead, the small terminal fragment containing Neo3 stabilized in three different ways, by forming a small acrocentric, by fusing with hamster chromosomes or by creating a small metacentric chromosome (Fig. 2, S6 and S7).

Very interesting is the clone observed in File S8, where a partial duplication of the signal produced by the distal probe (green signal) appears near the break site. It is likely that the duplication includes telomeric sequences to stabilize the broken chromosomal fragment, but further experiments are needed to verify it.

**Fig. 4:**
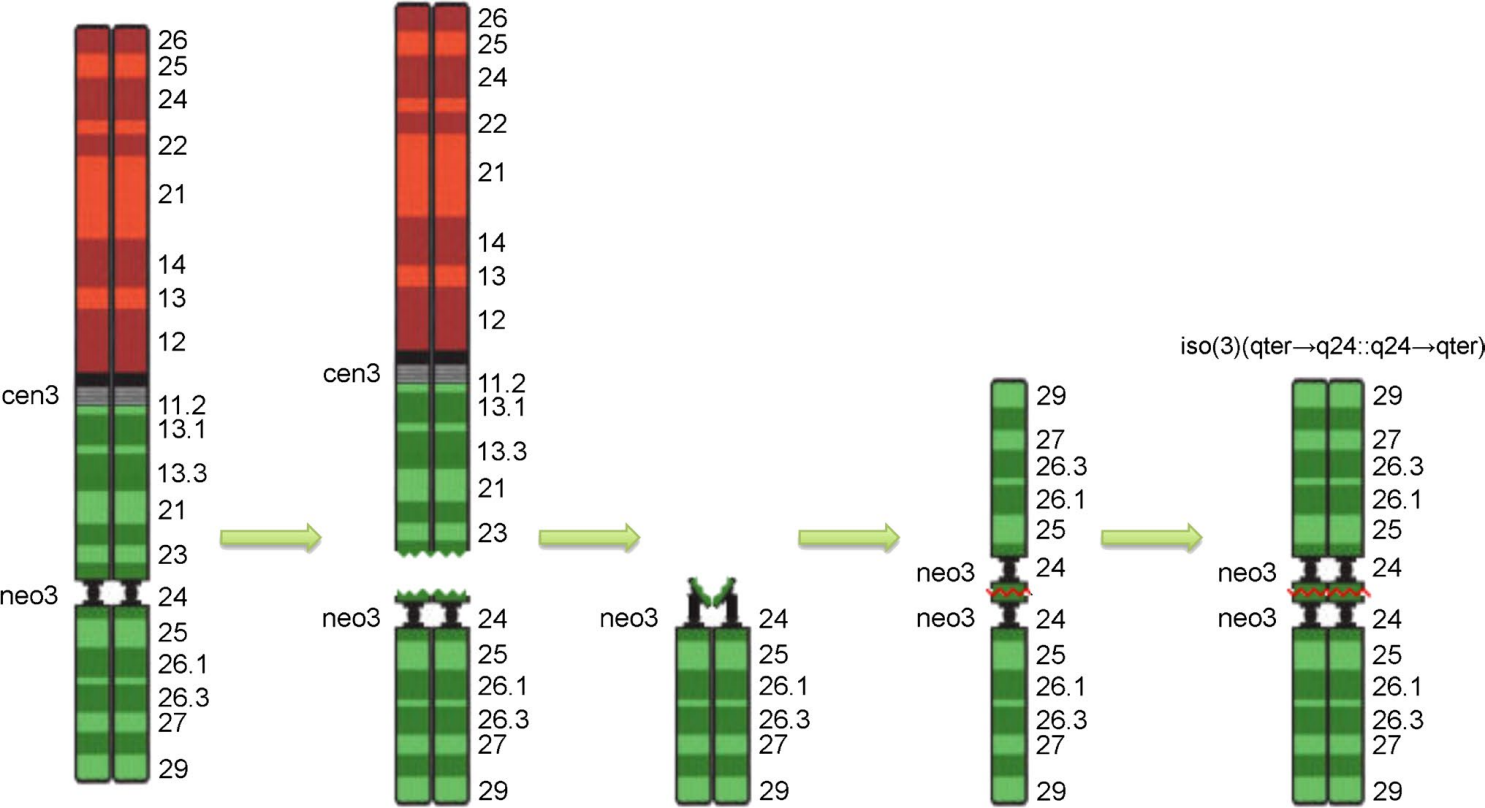
Model of isochromosome formation. Shown is a hypothetical model of the creation of the isochromosome containing two copies of the region 3q24 to qter, after the induced breakage of Neo3 by CRISPR-Cas9 methods

Detailed cytogenetic characterization of the small metacentric chromosome showed that it was, in fact, a newly formed pseudodicentric isochromosome with a functional centromere in which the terminal 3q composes both chromosomal arms (Fig. 2f, g). Isochromosomes form as a consequence of centromeric misdivision following a transverse division that separates the p and q arms (Wolff et al. 1996), as depicted in Fig. 4, which shows a model describing the mechanism leading to the derivative isodicentric chromosome we found.

Although being strongly negatively selected in vivo, isochromosome formation is far from rare in clinical cases, where they are associated to neoplasia (Mertens et al. 1994) and genetic disorder as Turner (Dalton et al. 1998) and Pallister-Killian (Izumi and Krantz 2014) syndromes. Interestingly, the specific occurrence of 3q sSMCs has been already reported (Barbi et al. 2003; Cunha et al. 2016; Gimelli et al. 2007; Izumi et al. 2008), and previous studies have proposed the presence of the BCL6 gene (3q27.3) as an explanation for the positive selection of cells containing multiple copies of this small fragment of the genome. This gene is, indeed, considered responsible for the acquisition of the cellular proliferative advantage seen in lymphomas (Batanian et al. 2006).

However, it has already been described in maize, for example, that an isochromosome was formed following a chromosome break (Douglas et al. 2021).

In conclusion, we have shown that the selective pressure exerted by a living organism or in vitro by a cell line is essential to rescue chromosome fragments derived from a double-stranded break.

We have also shown that with CRISPR/Cas9 technology it is possible to generate a pseudodicentric isochromosome in an in vitro system. This will be useful to build up cellular models for simulating the biological and pathological conditions in which isodicentric chromosomes are often observed.

## Supplementary Information

Below is the link to the electronic supplementary material.

Supplementary file1 (GFF 36878 KB)

Supplementary file2 (GFF 36880 KB)

Supplementary file3 (XLSX 15 KB)

Supplementary file4 Results of the T7E1 assay. Lane “-“ represent the not digested amplicon (300 bp band), while lane “+” indicates the digested one. The latter shows two bands at about 100 and 200 bp, respectively. (JPG 117 KB)

Supplementary file5 Immuno-FISH characterization of clone E8, containing the 3q terminal fragment fused with a hamster chromosome. Red probe: RP11-21N8; green: anti-CENPC antibody; blue probe: RP11-498P15. (JPG 171 KB)

Supplementary file6 FISH characterization of clone G8, containing the 3q terminal fragment stabilized by forming a small acrocentric chromosome containing a duplication of the q arm telomere on the p arm. Red probe: RP11-498P15c3; green probe: RP11-693H4; blue probe (no signal obtained): RP11-21N8C5. (JPG 98 KB)

## Data Availability

The datasets generated during and/or analyzed during the current study are available in the National Center for Biotechnology Information (NCBI) Short Read Archive (https://www.ncbi.nlm.nih.gov/sra), code PRJNA793381.
