# Supplementary figures and images for: Genome characterization and CRISPR-Cas9 editing of a human neocentromere
Source: Chromosoma. 2022 Aug 17;131(4):239–51. doi: 10.1007/s00412-022-00779-y (PMC9674717; doi:10.1007/s00412-022-00779-y)

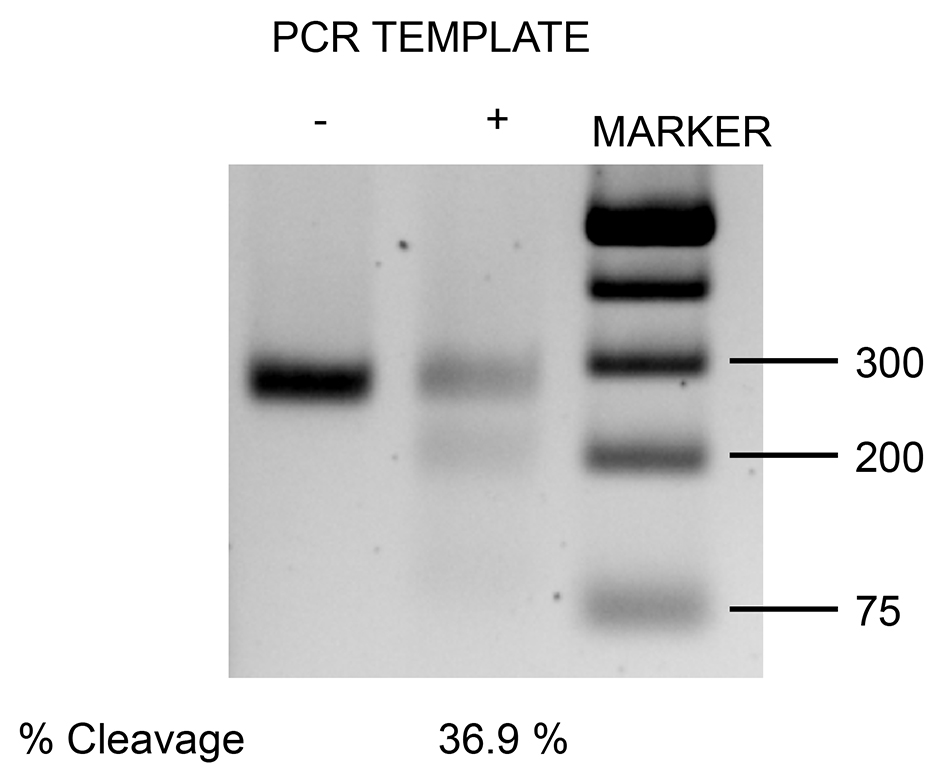

Supplement: Supplementary file 4 — Supplementary file4 Results of the T7E1 assay. Lane “-“ represent the not digested amplicon (300 bp band), while lane “+” indicates the digested one. The latter shows two bands at about 100 and 200 bp, respectively. (JPG 117 KB) [file 412_2022_779_MOESM4_ESM.jpg]

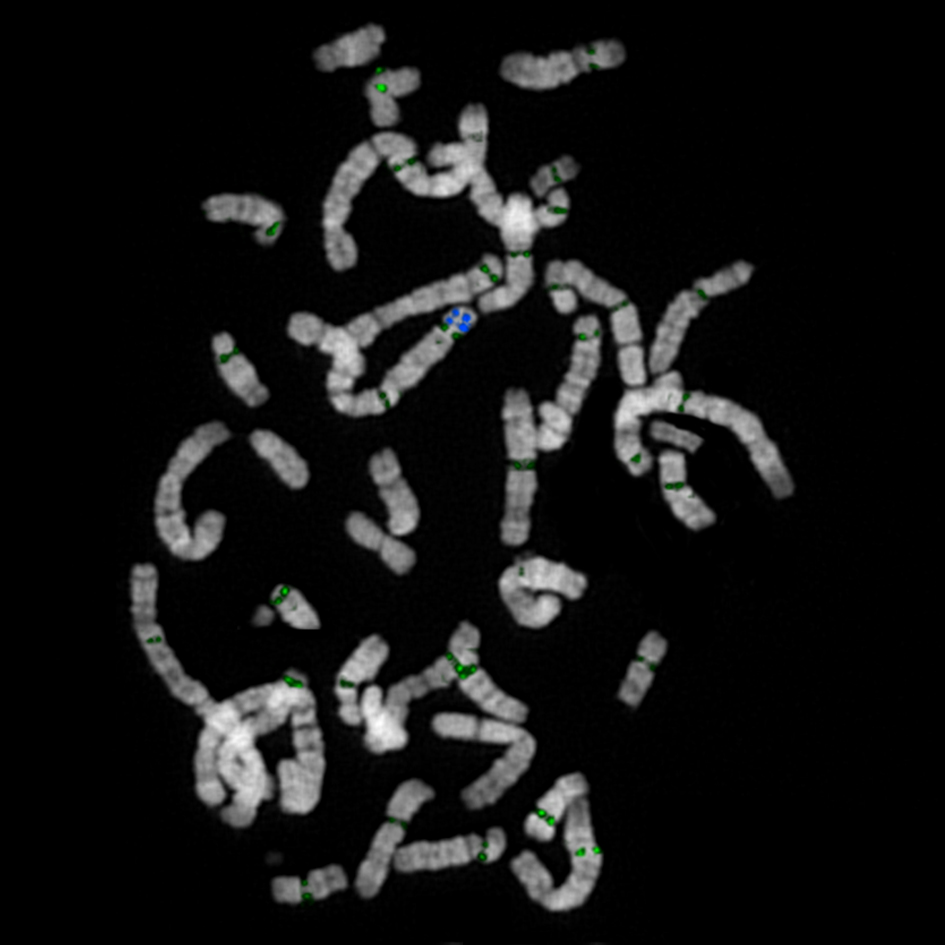

Supplement: Supplementary file 5 — Supplementary file5 Immuno-FISH characterization of clone E8, containing the 3q terminal fragment fused with a hamster chromosome. Red probe: RP11-21N8; green: anti-CENPC antibody; blue probe: RP11-498P15. (JPG 171 KB) [file 412_2022_779_MOESM5_ESM.jpg]

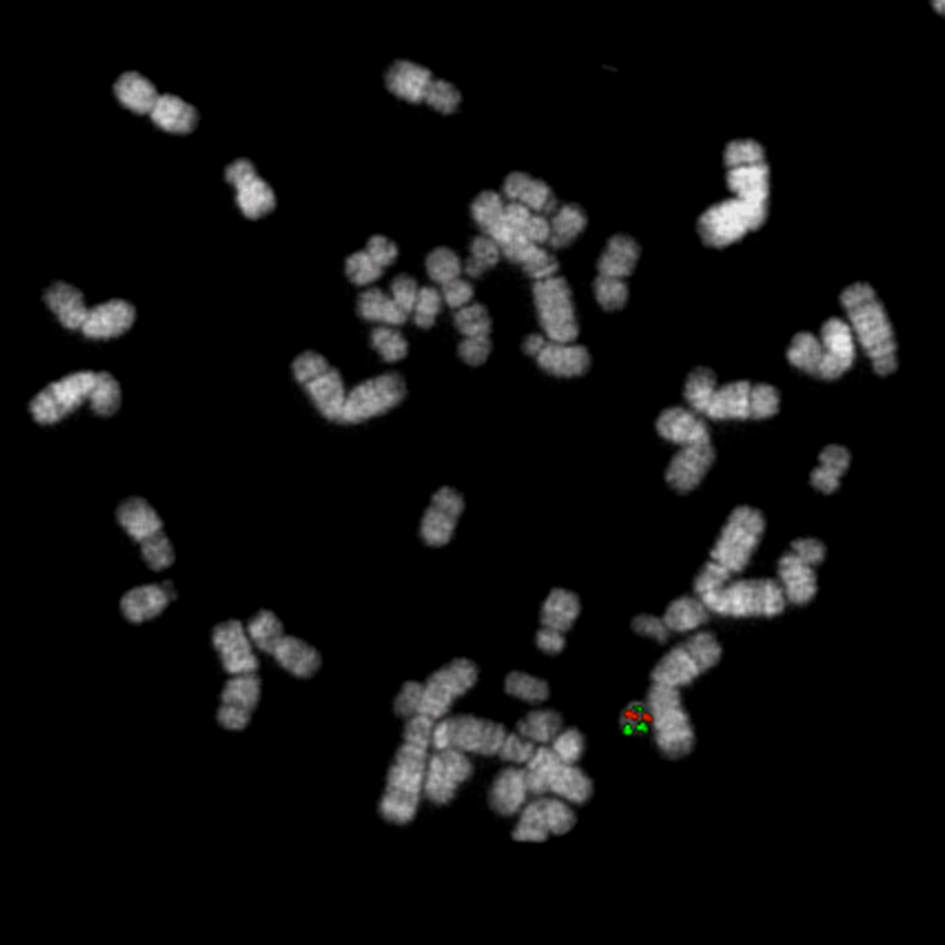

Supplement: Supplementary file 6 — Supplementary file6 FISH characterization of clone G8, containing the 3q terminal fragment stabilized by forming a small acrocentric chromosome containing a duplication of the q arm telomere on the p arm. Red probe: RP11-498P15c3; green probe: RP11-693H4; blue probe (no signal obtained): RP11-21N8C5. (JPG 98 KB) [file 412_2022_779_MOESM6_ESM.jpg]
